# Uncovering the mysteries of human gamma delta T cells: from origins to novel therapeutics

**DOI:** 10.3389/fimmu.2025.1543454

**Published:** 2025-04-10

**Authors:** Sylwia Biały, Katarzyna Bogunia-Kubik

**Affiliations:** Laboratory of Clinical Immunogenetics and Pharmacogenetics, Hirszfeld Institute of Immunology and Experimental Therapy, Polish Academy of Sciences, Wrocław, Poland

**Keywords:** γδ T cells, autoimmunity, diseases, novel therapies, treatment

## Abstract

Gamma delta (γδ) T cells represent a unique and distinct population of lymphocytes that bridge the innate and adaptive immune responses. This functional duality positions them as one of the pivotal elements in the evolution and development of the human body’s defense mechanisms. This review aims to provide a comprehensive and in-depth overview of γδ T cells, covering their origins, development, classification, and functional roles in immunology. Special attention is given to their involvement in the pathogenesis of autoimmune and cancer-related diseases—areas that remain subjects of intensive research with many unanswered questions. Additionally, this article explores the therapeutic potential of γδ T cells, which hold promise as a novel approach to treating various difficult-to-manage diseases. The review also presents an analysis of the latest clinical studies utilizing γδ T cells, emphasizing their emerging role in modern medicine. The ultimate goal of this work is to offer a holistic perspective on the current state of research on γδ T cells and their prospective applications in immunotherapy and cancer treatment, highlighting their potential to become a groundbreaking tool in future medical interventions.

## 1 Introduction

The documented history of γδ T cells begins in the 1980s, with the observation of a novel T-cell receptor (TCR) chain (1, 2). In 1987, scientists described an associated CD3 molecule - an unknown heterodimer comprising a chain generated by γ genes and a second chain termed δ chain (3). For over three decades, γδ T cells have been the subject of investigation by numerous researchers in different contexts.

Γδ T cells represent the initial group of lymphocytes formed in the thymus during ontogeny in all vertebrate species (4). Double-negative (CD4-CD8-, DN) thymocytes, serving as progenitor cells, initiate the formation of both αβ and γδ chains during the second stage of their maturation pathway (DN2). Fully developed TCR γδ are present in the third stage of thymocyte maturation (DN3) and it is at this juncture that the differentiation occurs between the αβ and γδ T cell lineages (5, 6). Some γδ lymphocytes leaving the thymus have the ability to perform specific anti-infective defense, which allows for a quick reaction in the newborn’s first contact with pathogens when classic T lymphocytes still acquire their abilities (7). Mature γδ lymphocytes constitute up to 5% of the total T lymphocyte population in adult humans and are distributed not only in the blood and lymphoid organs but also in various tissues such as the skin, lung and intestinal epithelium, and liver (8).

The interest in γδ T cells may arise from the unique combination of characteristics associated with both innate and adaptive immunity, positioning them as an evolutionary link in the development of these two defense mechanisms (9). Moreover, in contrast to classical T cells, γδ T lymphocytes do not necessitate antigen processing by antigen-presenting cells (APCs) and exhibit the ability to recognize non-classical antigens, while they are not under the major histocompatibility complex (MHC) restriction during antigen presentation. Possibly the mere connection of the γδ TCR receptor with the recognized antigen is sufficient for activation. The specific resemblance between γδ cells and natural killer (NK) cells, both functionally and in terms of surface receptors, can be another aspect of interest. Recognizing these shared characteristics provides a substantive basis for the development of innovative therapies for highly diverse disorders targeting these specific cell subsets (10, 11). However, a gap in knowledge about γδ T cells still persists, and its closure has the potential to reshape modern immunology.

## 2 γδ T cells – a whole range of variability

### 2.1 Essential signaling in thymocyte development

The process of γδ T cell development is observed in the thymus of all jawed vertebrates during ontogeny. Initial cells in the thymus originate from the bone marrow. Under the influence of Notch 1 (N1) and Delta-like 4 (DL4) signals, they give rise to a common thymic progenitor cell line called “early thymic precursors” (ETPs), serving as the starting population for both αβ and γδ T cell lineages (12). ETPs represent the first stage of thymocyte development from a hematopoietic stem cell. They are double-negative (DN; CD4-CD8-) lineage (DN1) characterized by the absence of CD25- expression and the presence of CD44+ on their surface. Subsequent developmental stages of thymocytes DN2-DN4 are distinguished mainly based on the detection of differences in the expression of these two receptors. CD117 is considered to be a factor enabling the survival of double-negative thymocytes and the distinction of individual thymocyte stages. Especially in the case of DN2 (CD117+CD25+CD44+) and DN3 (CD117low/-CD25+CD44low) stages, the differentiation might be difficult due to their apparent homogeneity (13, 14).

**Figure 1:**
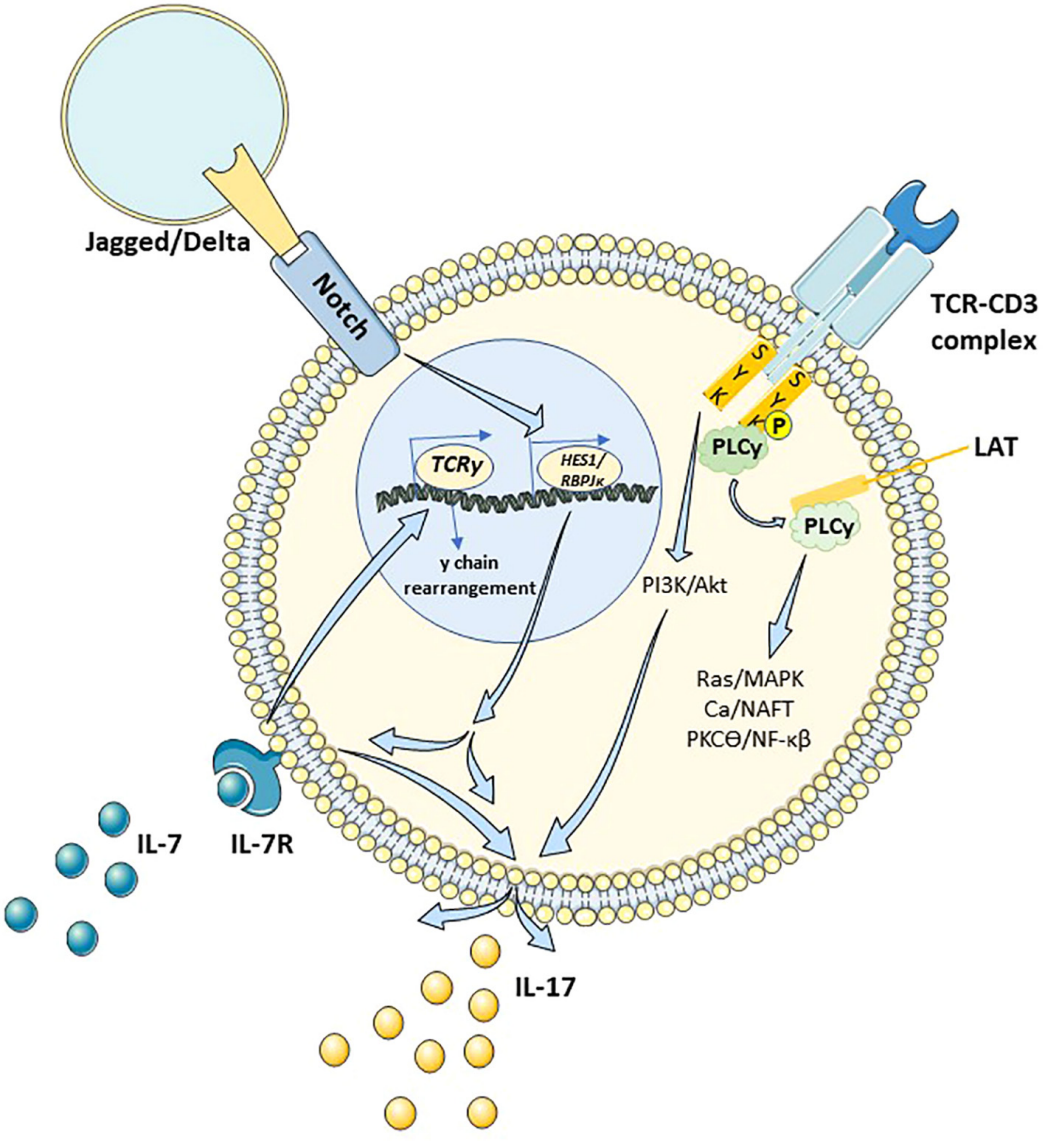
Schematic representation of γδ T cell and possible mechanisms involved in the regulation of its activity via engagement of TCR and Notch signaling pathways as well as IL-7 and IL-17 production. More detailed description is given within the manuscript text. TCRγ, T cell receptor gene γ chain; HES1, hairy and enhancer of split-1; RBPJκ, recombination signal-binding protein of immunoglobulin Jκ; Syk, spleen tyrosine kinase; PLCγ, phospholipase C-γ; LAT, linker for activation of T cells; MAPK, mitogen-activated protein kinases; NFAT, nuclear factor of activated T-cells; PKCθ, protein kinase C theta; NF-κB ,nuclear factor kappa B; IL-, interleukin 7; IL-7R, interleukin 7 receptor; IL-17, interleukin 17.

A crucial moment in determining the fate of ETPs is the induction of *IL2RA* gene expression, which encodes for the CD25 protein located on the thymocytes surface. This process occurs during thymocytes transition from the DN1 to the DN2 stage and is associated with the initiation of TCR receptor formation and differentiation. Between the DN2 and DN3 stages, gene rearrangement encoding individual TCR chains begins, including the γδ chains as well as the conventional β and partially α chains. The choice of lymphocyte differentiation towards αβ or γδ T is not fully explained, but two theories have originally attempted to elucidate it: 1) the stochastic model and 2) the instructive model (12, 15). In the stochastic model, progenitor cells can create γδ TCR receptors by rearranging *Tcrg* and *Tcrd* or create pre-TCR by rearranging *Tcrb*. This model assumes that TCR is not paramount in determining cell fate, but each cell belongs to a predetermined group. The mechanisms governing this process are unknown, but cells that do not develop a TCR compatible with their fate die. The second model explaining the determination of ETPs fates involves TCR and the possibility of tightly controlled transition of pre-TCR into the αβ receptor, while the presence of γδ TCR forms γδ T cells (15). Results and observations from various scientific teams led to the proposal of another model involving signal strength (16). Specifically, the paramount signal appears to originate from the Notch receptor family while it has been demonstrated that its activity regulates the expression of genes such as *Il2ra, Gata3, Bcll1b, Notch3*, and *Trca.* However, the activity of these genes is detected at various stages of thymocyte maturation (17). On the other hand, Notch signaling in thymocyte fate determination relies on cooperation with one of its ligands from the DL or Jagged family (**Figure 1**). The signal from DL4 simultaneously supports the generation of both αβ and γδ chains. A similar effect is observed with the combination of the Jagged2 ligand and Notch1 receptor, but this signal is weaker than the Jagged2 and Notch3 combination, which, in turn, promotes the formation of γδ chains and represses β chain differentiation (18). An interesting observation is that in a mouse model, the determination of T-cell fate, depending on Notch activity and signaling strength, is opposite to that in humans (19).

Furthermore, studies on mice have shown that the transition between successive stages of thymocyte development (from DN2 to DN3) requires not only Notch signals but also the involvement of interleukin 7 (IL-7) (20) (**Figure 1**). IL-7 and its receptor IL-7R are crucial for the development of γδ T cells and other lymphocyte lineages (21). Mice lacking IL-7R experienced a halt in the rearrangement of γ chain V-J segments, leading to the absence of γδ T cells (22, 23). Additionally, high levels of IL-7Rα and IL-7 contributes to the growth of the γδ T cell subpopulation capable of producing IL-17 (γδ17). Referring to the γδ17 T cell subpopulation again, it is worth mentioning the Notch signaling and its action through the hairy and enhancer of split-1 (HES1) transcription factor (24) or recombination signal-binding protein of immunoglobulin Jκ (RBPJκ) (25). Both pathways are independent of each other, and the inhibition of one pathway does not affect the intrathymic differentiation of γδ17 T cells by the other pathway (7). However, it is not the only signaling pathway supporting the generation of γδ17 T cells. Another pathway involves the spleen tyrosine kinase (Syk) receptor. On one hand, the signalosome called the Linker for activation of T cells (Lat), the main target for Syk in γδ cells, leads to the activation of the mitogen-activated protein kinases (MAP) cascade, as well as pathways involving nuclear factor of activated T-cells (NFAT) and the nuclear factor kappa B (NF-κB), influencing the induction of γδ T cells (26). On the other hand, there is a Lat-independent pathway where Syk directly interacts with the regulatory subunit of Phosphoinositide 3-kinase (PI3K), activating the PI3K/Akt axis (26) (**Figure 1**). Signals of moderate strength from this pathway are also a crucial element in the differentiation and maintenance of activity in γδ T cells producing IL-17 through the maintenance of the expression of key regulators such as retinoid-related orphan receptor (RORγt) and musculoaponeurotic fibrosarcoma oncogene homolog (c-Maf) (27, 28). It has been shown that both overactivation and downregulation of the PI3K/Akt axis reduce the development of γδ17 T cells (28) (**Figure 1**).

The process of ETPs fate determination in γδ T cells is highly complex, and despite numerous studies, there are still unresolved gaps in the signaling network, leaving a wide field for further research.

### 2.2 Creation waves

In the human fetal life, the second site for γδ T lymphocyte generation is the liver. This subset of cells generated in the fetal liver constitutes approximately 30% of all CD3+ lymphocytes. These cells are described as CD4+ and simultaneously CD8-, which is atypical for cells originating from blood and thymus. Studies in mice have shown that γδ cells in the fetal liver can undergo their own thymus-independent developmental cycle. It has been found that murine liver hematopoietic progenitor cells, described as Lin-negative and Sca-1 and Mac-1 positive (LSM), differentiate into precursors of γδ T cells or γδ TCR cells under experimental conditions. Interestingly, these cells are not capable of producing IL-17 but produce IFN-γ (29).Additionally, a consistent motif Vγ9 has been observed in human liver γδ cells along with a preferred chain of Vδ2 and Vδ3. The Vγ9Vδ2 subset was detected in the fetal liver as early as 5-6 weeks of gestation, becoming the dominant subpopulation around 20-30 weeks (8, 30).

Γδ T lymphocytes are produced in distinct waves during murine fetal life. The model of waves is based on observable changes in the functions of γδ cells, such as cytokine secretion or TCR γδ characteristics. The model established for murine development has been attempted to be applied to human fetal development (31). The first wave in humans is considered to be the pre-thymic subpopulation Vγ9Vδ2 formed in the fetal liver (5-6 weeks of gestation) (30). This assumption is supported by the fact that human thymus organogenesis begins around 6-7 weeks of gestation, and the first γδ thymocytes were detected around 10-11 weeks of gestation, indicating the pre-thymic origin of the first wave. Fetal γδ T cells of thymic origin arise in a slightly shifted wave compared to the liver wave. Fetal thymic γδ T cells mainly contain the Vδ2 chain with low variability. Additionally, fetal thymic cells, both Vδ2 and present in small amounts Vδ1, are effector-programmed and possess characteristic fetal CD3γ and CD3δ sequences, absent in postnatal γδ cell waves. While Vγ9-Vδ2+ cells dominate in the fetal thymus, the Vγ9Vδ2 cell group is the predominant subpopulation in fetal peripheral blood. However, it is gradually replaced by the subsequent wave of Vδ1 cells, which peak in umbilical cord blood at birth (32).

### 2.3 Rearrangement of TCR γδ, mandatory classification and γδ selection

The rearrangement of the TCR γδ initiates in the thymus at the DN2 stage of thymocytes, marked by the presence of CD44 and CD25, likely occurring in parallel with *Tcrb* rearrangement but preceding the rearrangement of the α chain of the classical TCR (33). The primary objective of all TCR receptor groups is the capacity for efficient antigen recognition, particularly those potentially harmful to the body. This necessitates a broad repertoire of receptor sequences capable of antigen recognition. The prevailing opinion is that, at any given time, the human body harbors a greater diversity of TCRs than the quantity of circulating lymphocytes (34). Several mechanisms ensure such diversity. Firstly, all genes encoding TCR receptor chains have a segmental structure, consisting of variable (V), diversity (D), joining (J), and constant (C) regions, with *Tcra* and *Tcrg* genes lacking D segments. The segments are randomly combined by excising the DNA fragment between the joined segments. According to the IMGT/GeneInfo database, humans possess 6 functional Vγ segments and 3 mere Vδ segments, as well as 5 shared with Vα locus (35–37). There are more V regions in the *Tcra* and *Tcrb* genes, allowing for the generation of a larger number of classical TCR combinations. However, mechanisms that significantly increase the diversity of TCR γδ include the presence of additional D regions in *Tcrd*, random deletions and insertions of nucleotides (named N), or the insertion of palindromic sequences (named P) during the V(D)J region joining (38). Nucleotides are added through terminal deoxynucleotidyl transferase (TdT) (39), generating variability at the junctions. One of the crucial components interacting with the antigen is complementary determinant regions (CDRs), responsible for the specificity of the V region. Among them, the CDR3, arising from the combination of V, (D), and J segments, exhibits the greatest variability and is considered a key element in the diversity of TCR repertoire (38). The CDR3 in the δ chain is longer and more diverse compared to the classical β chain, exhibiting greater sequence variability, which enables γδ TCR to recognize both protein and non-protein antigens (40).

The nomenclature used in describing γδ T cell subgroups is based on the presence of specific V segments in γ and δ chains. Initially, the 14 loci for Vγ genes and pseudogenes allowed classification into 6 major cell subpopulations. The largest group was VγI, encompassing the majority of functional genes (V2, V3, V4, V5, V8). The V9 loci formed a distinct VγII group (41). Currently, the prevailing nomenclature does not consider such divisions and relies on names derived from the variants of V segments in combined chains. Nevertheless, TCRs possessing Vγ9 are still referred to as VγII or Vγ2 in some works, which can be misleading. Nowadays γδ T cells are divided according to the V segments in the δ chain into Vδ1 and Vδ2 as well as Vδ3 group. The combination of selected γ and δ chains is characteristic for specific tissues (42) and the updated nomenclature follows a system more in line with the naming proposed by Heilig and Tonegawa, without subdividing the groups as in Lefranc and Rabbitts’ nomenclature. This approach has been adopted in the present work (41). According to this, the Vδ2 chain is most commonly associated with Vγ9 (Vγ9Vδ2) and constitutes the main subset of circulating γδ cells in healthy human peripheral blood (43). For comparison, the Vδ1 chain is associated with a greater number of γ chain variants and represents the main population inhabiting the liver (Vγ4), skin (Vγ4, γ5, γ6), spleen (Vγ4), or epithelium (Vγ7) (42). Additionally, the ratio between Vδ1 and Vδ2 cells in the blood varies not only in infectious and cancer diseases (44, 45) but also in human individual development. Starting from the embryonic stage, Vδ2 cell production dominates, then, at birth, there is a drastic decrease in the production of all γδ T lymphocyte groups. Around the age of 30, there is a significant dominance of Vδ2 cells over Vδ1, followed by a levelling off around the age of 45, with the Vδ1 subpopulation dominating in the proportions in the later years of adult life (45, 46). In tissues, this ratio changes even earlier, as the Vδ1 population already dominates at birth (47).

### 2.4 Untypical way of antigen recognition and co-stimulatory molecules

T lymphocytes bearing the classical TCR αβ receptor recognize foreign antigens of bacteria and viruses located both extracellularly and intracellularly after the incorporation of the foreign antigen into the membrane of the infected cell. This recognition is facilitated by antigen-presenting MHC molecules, a name retained to underscore that these were the first known group of proteins crucial in the graft acceptance process by the recipient’s body. An additional element enabling antigen recognition is provided by co-receptor proteins CD4 and CD8 on the surface of T lymphocytes αβ, allowing for the recognition of different classes of the MHC complex (class II and class I, respectively). As this information is considered fundamental for immunology understanding, it may come as a surprise that it does not apply to T γδ lymphocytes, which have developed their own rather sophisticated recognition system. Among The groups taking part in antigens recognition, and stimulation of γδ T cells are MHC, MHC-like molecules, the B7-like molecule family, Ig-like molecules, as well as NK cells-characterized receptors.

#### 2.4.1 MHC molecules

MHC molecules relatively rarely serve as direct ligands for γδ TCR. However, they have been described in both mice (IEk and I-Ad) (48) and in humans, where in some circumstances the ability to recognize classical HLA molecules such as HLA-A24 (49), -B27 (50), -A2 (51), as well as non-classical HLA-E (52) has been defined. On the other hand, the lack of requirement of MHC restriction was demonstrated through studies in mice with a knockout for β2-microglobulin (β2-M), an integral part of the HLA molecule that is essential for revealing the specificity of MHC antigens. In this mice model, there was a minimal presence of T αβ lymphocytes due to an inhibited positive selection process in the thymus. On the other hand, it was shown that the lack of β2-M practically does not alter the parameters concerning T γδ cells (53, 54). Despite those results, it is hypothesized that a small subset of T γδ lymphocytes depends on MHC restriction. However, their overall abundance is low enough that the elimination of these cells in the β2-M knockout model falls below the detection threshold in the T cells’ entire population (55).

#### 2.4.2 MHC-like molecules

Other studies in mice have identified proteins referred to as MHC class I-like, capable of stimulating T γδ lymphocytes. The most well-characterized protein in this group is polyphemusin II peptide (T22). Structurally, this peptide partially resembles MHC (56), yet it lacks the ability to bind the presented antigen, thereby activating up to 1% of T γδ lymphocytes in normal mice (57, 58). This process is primarily achievable due to the presence of a conserved fragment containing the sequence W….EGYEL in the loop determining the complementarity of the CDR3δ of the TCR γδ receptor. The linkage of aminoacids between tryptophan (W) and the EGYEL motive is variable in length and chemical nature, which may affect the binding of TCR with T22. Nevertheless, Sandstrom et al. have identified similarities in the binding of these molecules to the classical MHC and TCR αβ molecule binding scheme (58). Initial studies aiming to establish the importance of T22 in T cell development yielded conflicting results (59–61). However, the creation of a mouse model with a specific knockout for T10 and T22 demonstrated that while T22 is a key factor in TCR γδ development, it is possible to generate a small number of slightly impaired cells, yet still reactive to T22 without this antigen stimulation (62).

In humans, γδ T cells are capable of recognizing MHC-like molecules from the MIC family (MICA and MICB). These molecules are highly polymorphic and serve as stress-induced ligands for immune cell activation. Importantly, γδ T cells can recognize MIC molecules independently of the present allele, and in a relatively direct manner, as MIC molecules do not function like classical MHC since they do not present peptides to T cells. It must be mentioned that the recognition of MIC molecules does not involve the CDR3 fragment of the TCR chain (63). In this case, the role of a specific co-receptor is significant, with NKG2D serving as the receptor that recognizes these molecules. NKG2D is characteristic for several types of cells such as NK cells or CD8+ αβ T cells, but also present in abundance on the surface of γδ T cells (64).

#### 2.4.3 B7-like molecules

Molecules related to the B7 family including butyrophilins (BTN) can also act as ligands for γδ TCR. It has been demonstrated that butyrophilin 3 A1 (BTN3A1) plays a crucial role in recognizing phosphoantigen by TCR Vγ9Vδ2 (65), especially in the context of cancer cell recognition, where, in some but not all cases, cell-to-cell contact is required (66–68). This suggests that BTN3A1 acts as an antigen-presenting molecule. While the intracellular B30.2 domain of BTN3A1 has been previously recognized for its significant role, the mechanism of cooperation between BTN3A1 and the TCR γδ was elucidated by Sandstrom’s team in 2014 (69). The current theory explaining the involvement of BTN3A1 in the activation of γδ T cells suggests binding of the phosphoantigen by the intracellular B30.2 domain and the formation of a complex with BTN2A1, causing structural changes in the molecule and transmitting the signal to the TCR γδ. Interestingly, this process involves two distant binding sites on the TCR γδ. The BTN2A1 molecule binds to the Vγ9 region, and possible binding sites for BTN3A1 are located in the CDR2 and CDR3 loops (70). Numerous supporting proteins are involved in this process (55). Nevertheless, the precise course of the interaction between BTN3A1, the antigen, and TCR γδ remains a subject of research and requires further exploration (71). However, there is already discussion about the role of butyrophilin in Vγ9Vδ2 T cell-targeted immunotherapy (72).

Recent studies highlight the intriguing role of BTNs and butyrophilin-like (BTNL) proteins in the selection and maintenance of γδ T cells. In the murine intestine, Btnl1 and Btnl6 heterocomplexes, expressed on the surface of enterocytes, regulate the maturation and expansion of Vγ7 T cells, promoting their phenotypic transition into mature γδ T cells (73). Similarly, in the human intestine, BTNL3 and BTNL8 complexes, expressed by intestinal epithelial cells, shape the organ-specific repertoire of Vγ4 T cells, influencing their selection and function (74). Disruption of functional BTNL3/BTNL8 complexes impairs the selection of Vγ4 T cells expressing CD103, a key marker of intraepithelial lymphocytes (IELs). Notably, inflammatory bowel diseases (IBD) have been associated with an overall reduction in γδ T cells, with a disproportionate decrease in the cytotoxic Vγ4 CD103+ subset. These cells exhibit high expression of activation receptors such as NCR1 and NKG2C (75), suggesting their potential therapeutic relevance. Moreover, enterocyte-expressed BTNL molecules support the long-term survival of γδ T cells in the intestine, independent of microbiota or peripheral lymphoid organs (76).

The effect of B7 family members in the activation of γδ lymphocytes also occurs through surface receptors known as Ig-like receptors. An example of such co-signaling is the interaction of the B7.1 and B7.2 proteins (commonly referred to as CD80 and CD86, respectively) located on APCs with the Ig-like receptor on γδ lymphocytes, in this case, the CD28 protein. Despite previous conflicting results regarding changes in the expression of the CD28 receptor on γδ cells (77, 78), Ribot and colleagues demonstrated a significant role of this receptor in the co-activation of γδ cells. They showed that the signal from CD28 influences the production of IL-2 and as a result of these events, human γδ cells, as well as those in the murine model, exhibit increased proliferation and survival capacity (79).

#### 2.4.4 Ig-like co-receptors

Ig-like receptors are also involved in the recognition of cancer cells. An example of such action is demonstrated by DNAX accessory molecule-1 (DNAM-1). Phosphorylation of Ser329, one of the three possible phosphorylation sites on the intracellular domain of this protein, activates a cascade of kinases, leading to signal transmission. Studies have shown that blocking the activity of DNAM-1 results in a lack of cytotoxic response against cancer cells (80, 81).

#### 2.4.5 NK-specific receptors

The last mentioned group of receptors are primarily associated with NK cells. This category includes receptors from the natural killer group 2 (NKG2), natural cytotoxicity receptors (NCR), and killer cell immunoglobulin-like receptors (KIR) groups. These are also abundant in γδ T cells and participate in the recognition of infectious antigens and tumor-transformed cells, as well as in the generation of cytolytic reactions against them (82, 83).

Among the NKG2 molecules, the best described in the context of γδ T cells appears to be the activating receptor NKG2D. Among the γδ cell population residing in human blood, the expression of NKG2D is approximately 10 times lower compared to NK cells (84). Stimulation of γδ cells through NKG2D occurs independently of the TCR and resembles the process that occurs in NK cells upon receptor binding to ligands such as MHC-like molecules: MICA/B. Most likely, the signaling pathway in γδ cells proceeds through the adaptor protein DAP10 and then engages the kinase PI3K (85, 86). It has been shown that in mice, signaling from this pathway is sufficient to induce cytolytic reactions originating from dendritic epidermal T cells (DETCs), which constitute a subpopulation of γδ cells in the epidermis (87). Another interesting aspect is Ca^2+^ ions, which are considered essential for the effective proliferation and activity of cells in the immune system, thus serving as informational and activating elements for the cell (88). It has been demonstrated that simultaneous activation of γδ TCR and NKG2D causes a sudden and short-term influx of Ca^2+^. This is a characteristic model for effector memory T cells. The authors of the mentioned studies suggest that the course of cell stimulation influences the effector functions of Vγ9Vδ2 subpopulations (89). The phenomenon of Ca^2+^ ion influx in response to NKG2D activation may be additionally inhibited by the protein kinase C theta (PKCθ) inhibitor, indicating that it is a signal supporting the action of NKG2D (90). In light of these events, it is apparent that stimulation by the NKG2D receptor enhances the function of γδ T cells and boosts the cytotoxic response against foreign cells.

NCRs receptors are not constitutively present on γδ T cells but rather they are expressed in special environments, after TCR stimulation (90). Among the NCR receptors, the activating NCR2 (NKp44) has been most closely associated with γδ T cells. The presence of NCR2 on this non-classical cell subset was identified at the beginning of the 21^st^ century (91, 92). Furthermore, it has been demonstrated that blocking NCR2 limits cytotoxic capabilities against myeloma cells (92). NCR3 (NKp30) has also been detected on the Vδ1 subset, while NCR1 (NKp46) is found on the cytolytic intraepithelial Vδ1 T cell subset residing in the human intestines (93). Although the expression of these receptors is detected on specific γδ cell clones, the presence of each receptor is associated with the cytolytic abilities of the cells (94, 95).

There is very little literature on KIR receptors present on γδ T cells. However, it is known that similar to NK cells, KIR receptors on γδ T cells serve an inhibitory function. KIR receptors are present on Vγ9Vδ2 T cells capable of cytolytic reactions, whereas they are not detected in non-cytolytic cells. The Vγ9Vδ2 T cells subset with KIR receptors inhibits the lysis of tumor B cells possessing MHC class I (83). Additionally, blocking the interaction between KIR and MHC molecules activated the ability to recognize self-markers of γδ T cells and induced auto-reactivity (83). This is certainly an intriguing and poorly understood group of receptors located on γδ T cells, which requires investigation due to its therapeutic potential in combating both cancerous and autoimmune diseases.

In addition to the receptor groups outlined above, γδ T lymphocytes possess a wide repertoire of receptors that enable the characteristic integration of innate and adaptive immune response functions. This characteristic trait also renders γδ T cells with dual roles in immunological diseases: beneficial and adverse.

## 3 Dual role of γδ T cells in immune response

It is believed that γδ T cells combine characteristics of cells involved in both innate and adaptive immune protection (96). Additionally, they possess receptors on their surface that can both inhibit and activate their functional activity. Consequently, γδ T cells can be expected to participate in pathological conditions. Research supports this notion by indicating the involvement of γδ T cells in both defensive and reparative processes of the body as well as in promoting pathological states. Activated γδ T cells are capable of producing chemokines, growth factors, and cytokines characteristic for different Th lymphocyte subpopulations (97, 98), while they also possess cytotoxic abilities (92), and play a helper role for B lymphocytes (99).

Γδ T cells effectively regulate homeostasis in the tissues and organs where they reside, however, when extensively or inadequately stimulated they can turn against the host itself. Examples include pulmonary epithelial γδ T cells, intraepithelial lymphocytes (IELs) and γδ T cells in the epidermis of the intestine (named dendritic epidermal T cells - DETCs). These cells naturally participate in promoting the production of keratinocytes and growth factors for wound healing and maintaining the integrity and immunity of mucous membranes (100–102). Studies in mice have shown that overproduction of the transcription factor early growth response 3 (Egr3) stimulates the secretion of pro-inflammatory IL-17 by γδ T cells in the lungs, leading to pulmonary fibrosis (103). On the other hand, a deficiency of IL-17 in the lungs of the mouse model caused by limited stimulation from tumor necrosis factor α (TNF-α) weakens the inflammatory response, posing a danger in infectious and pathological processes (104). In the mouse intestine, TNF-α can disrupt the migration of IELs, potentially resulting in acute inflammation (100). Conversely, mice lacking IELs develop shorter intestinal crypts and poorer mucosa, resulting in less effective mucosal defense (105). Similarly, in mice with skin burn wounds, DETCs have been observed to regulate the influx of pro-inflammatory molecules to the injured site (106).

The dual nature of γδ T cells underscores their pivotal role in immune regulation, encompassing both protective and pathological processes. Understanding the complex internal and environmental mechanisms that govern their function is essential to harness their benefits while minimizing adverse effects.

## 4 Autoimmune diseases

One of the first publications pointed to the important role of γδ T cells in autoimmunity comes from the early nineties (107). Currently, it is known that γδ T cells play a significant role in the pathogenesis of autoimmune diseases, particularly in those affecting connective tissue. In connective tissue diseases such as rheumatoid arthritis (RA) and systemic lupus erythematosus (SLE), γδ T cells contribute to both protective and pathological processes. Their ability to produce a wide range of cytokines and chemokines positions them as key players in the inflammatory cascade, influencing the recruitment and activation of other immune cells. Understanding the mechanisms by which γδ T cells influence autoimmune responses is crucial for the development of targeted therapies aiming mitigation of γδ T cells’ detrimental effects while enhancing their protective roles. This chapter delves into the complex functions of γδ T cells in autoimmune diseases focusing on connective tissue disorders.

### 4.1 Rheumatoid arthritis

Initial reports on the role of γδ T cells in RA date back to the late 1980s and early 1990s (108). These studies indicated alterations in the levels of γδ T cells in the blood, synovial fluid, and synovial tissue of RA patients compared to healthy individuals (109). Additionally, variations in cell levels were observed in relation to C-reactive protein (CRP) concentrations in patients (110), as well as when dividing patients according to their age (111, 112). It was noted that RA patients with disease onset before the age of 45 exhibited a greater increase in γδ T cell numbers than older patients. This was attributed to the still undetermined repertoire of these cells in younger individuals. Hassan et al. hypothesized that antigenic stimulation during RA development leads to a sudden clonal expansion and a predisposition to a more aggressive disease course in younger patients, while older individuals with a defined repertoire of γδ T cell clonotypes do not respond as vigorously to RA-associated antigens (111).

Furthermore, the description of changes in HLA-DR expression on γδ T cells appears significant in the context of RA pathogenesis, with an increase in the percentage of HLA-DR+ γδ T cells in the peripheral blood of patients with active disease (113). It is now known that HLA-DR, particularly the HLA-DRB1*04 variant, plays a crucial role in RA pathogenesis by presenting extracellular antigens (e.g., bacterial or viral protein fragments) to helper T (CD4+) cells. The so-called shared epitope (SE) present in HLA-DR molecules predisposes to RA development by preferentially binding autoantigens, thereby leading to the activation of autoreactive CD4+ T cells and the initiation of an autoimmune response (114). In light of these facts, it can be inferred that γδ T cells significantly contribute to autoimmune response in RA (115).

Additionally, it has been demonstrated that immunization with mycobacterial heat shock protein 65 kDa (HSP65) causes an increase in the number of HLA-DR+ γδ T cells in the synovial fluid of RA patients (116). Stimulation of Vγ9Vδ2 T cells by isopentenyl pyrophosphate induces, among other effects, the production of IL-17 by these lymphocytes (117).

**Figure 2:**
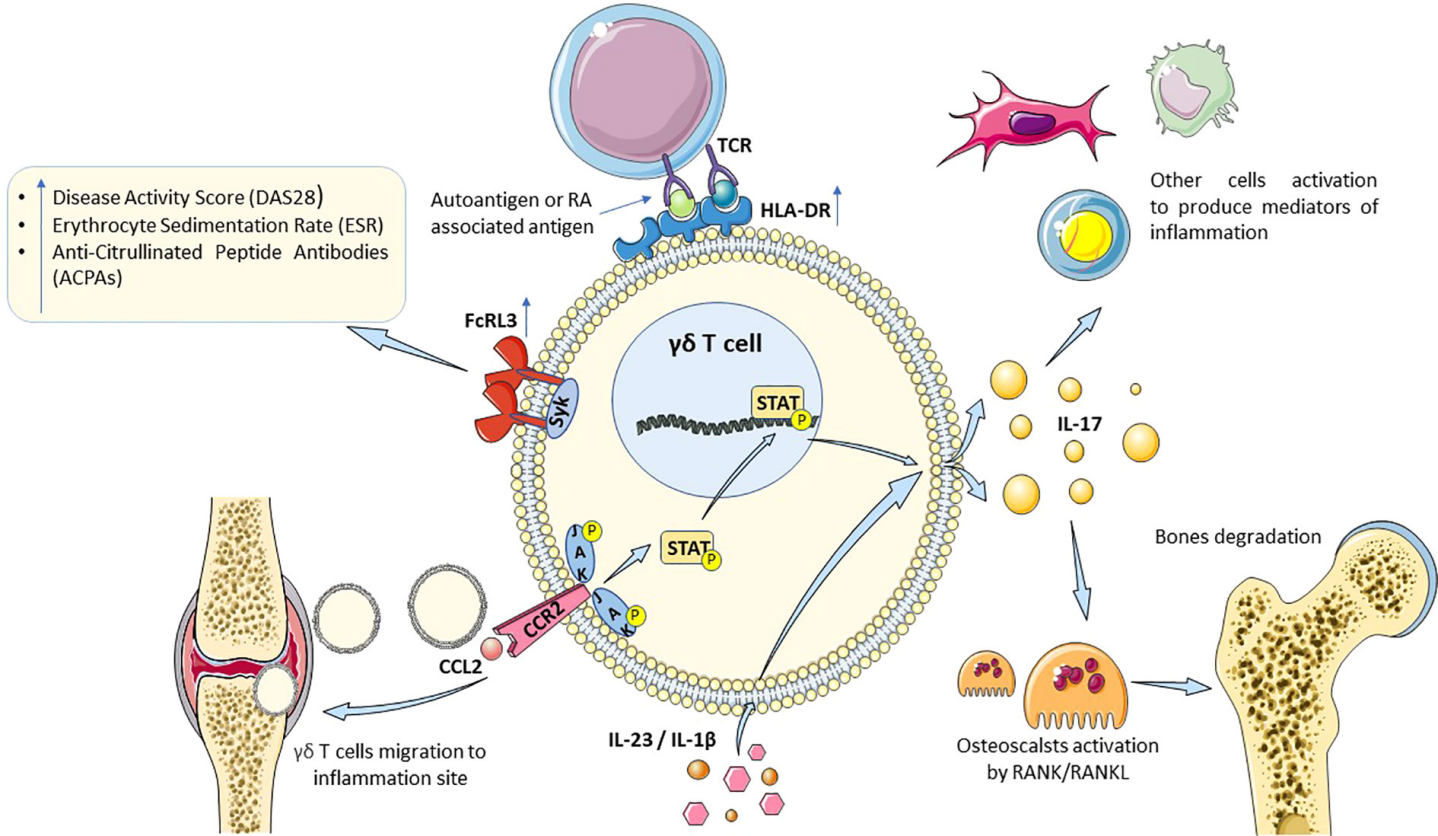
A proposed mechanism contributing to the pathogenesis of RA (rheumatoid arthritis), incorporating elements present in γδT cells and with proven pathogenic effects in T lymphocytes: T cell activation (HLA-DR expression), FcRL3 expression and involvement of Syk pathway, proinflammatory cytokine production including IL-17 leading to osteoclast activation and chemokine secretion facilitating γδ T cell migration to RA inflamed joints. More detailed description is given in the text. RA, rheumatoid arthritis; HLA-DR, human leukocyte antigen, DR isotype; IL, interleukin; RANK/RANKL, activator for nuclear factor κB (ligand); CCL2, chemokine (C-C motif) ligand 2; CCR2, C-C chemokine receptor 2; JAK, none-receptor tyrosine Janus kinase; STAT, signal transducer and activator of transcription protein; Syk, spleen tyrosine kinase; FcRL3, Fc receptor-like protein 3.

Th17 cells are the primary source of IL-17, a key pro-inflammatory cytokine in the pathogenesis of rheumatoid arthritis, while γδ T cells, differentiated into the γδ17 T subpopulation, play a secondary but still significant role in its production. Γδ T cells can produce IL-17 following stimulation by other molecules, such as IL-23 or IL-1β (118) (**Figure 2**). Classically, IL-17 contributes to the propagation of inflammation by stimulating various cell types, such as fibroblasts, macrophages, and chondrocytes, to produce other pro-inflammatory cytokines and chemokines. Those act as chemoattractants, recruiting neutrophils and macrophages to the site of inflammation, which is in most cases the joint. Moreover, IL-17 significantly contributes to the degradation of bone tissue by enhancing the expression of the Receptor Activator for Nuclear Factor κB (RANK) and its ligand (RANKL) and by activating osteoclasts (119) (**Figure 2**).

In the context of local inflammation occurring in the joints of RA patients, the migration of γδ17 T cells to the diseased site is crucial. During differentiation, γδ T cells exhibit increased expression of molecules CCR6, CCR2, and CXCR6 (120). CCR6 is present on classical Th17 lymphocytes and interacts with CCL20, causing Th17 migration to the site of inflammation (121) (**Figure 2**). For γδ T17 cells, the CCL2-CCR2 axis appears to be similarly important, facilitating their attraction to the inflamed joints (122, 123). The binding of CCL2 to its receptor can initiate, among other things, the JAK/STAT signaling pathway, the inhibition of which is targeted by biological therapies in the treatment of RA (124). In a mouse model of RA, treatment with Tofacitinib, which inhibits JAK1 and JAK3, and to a lesser extent JAK2 and TYK2, altered the levels of IL-17 and γδ17 T cells while promoting the presence of γδ T regulatory cells (125). Another mice study indicated that administration of ES-62 (a phosphorylcholine derivative molecule) lowered IL-17 levels in both joints and bones, whereas diseased mice showed an increased percentage of γδ17 T cells. *In vitro* studies demonstrated that ES-62 inhibits both γδ T lymphocytes and the dendritic cells (DCs) essential for modulating their both γδ T cells and DCs functionality (126).

Another potential mechanism determining the pathological processes in RA via γδ T cells is the high expression of the Fc receptor-like protein 3 (FcRL3) on their surface in RA patients. The increased expression of this receptor correlates with the Disease Activity Score (DAS28) and Erythrocyte Sedimentation Rate (ESR) (127). Furthermore, FcRs, and likely FcRL due to their significant homology, interact with Syk via its immunoreceptor tyrosine-based activation motif (ITAM) domain (127, 128), as well as its close homolog Zeta-chain-associated protein kinase 70 (ZAP-70) (127). Syk is essential for the development of the γδ T cell population. Elevated levels of Syk have been observed in RA patients and have been associated with higher titers of anti-citrullinated peptide antibodies (ACPAs) (129) (**Figure 2**). Mice deficient in both FcR and Syk do not develop collagen-induced arthritis (130), indicating the critical role of this pathway in the pathogenesis of RA (131).

In summary, γδ T cells contribute to the pathogenesis and progression of RA through both direct actions, such as the production of inflammatory mediators, and indirect mechanisms, including the activation of intracellular processes that create a pro-inflammatory environment conducive to joint inflammation and tissue destruction. However, further research is required to elucidate the precise role of these lymphocytes in RA (**Figure 2**).

### 4.2 Psoriasis

Psoriasis is a chronic autoimmune inflammatory skin disease characterized by red, scaly patches on the surface of the body. The etiology of psoriasis is complex, involving both genetic and immunological factors. Current research suggests that the dysregulation of T cells, including γδ T cells, plays a crucial role in the development and maintenance of inflammation in psoriasis. Studies on the role of γδ T cells in human psoriasis have revealed changes in the proportion of these cell subpopulations. Specifically, Vγ9Vδ2 T cells were found to be more abundant in the skin of affected individuals, while less frequent in the blood compared to healthy controls. This suggests a possible migration of these cells and their potential association with the disease (132). However, it is worth noting that role of γδ T cells in psoriasis are largely based on mouse models, which are extensively described in this chapter.

Numerous studies have shown that among the subpopulations of murine γδ T cells, the Vγ4 subset residing in the dermis is most closely associated with psoriasis, constituting approximately 20% of all γδ T cells in this tissue (133). Dermal γδ T cells differ somewhat from the epidermal DETC subpopulation, particularly in the structure of their TCR receptors. While epidermal T cells express the Vγ5 chain, this chain is rarely found in dermal T cells (134). Dermal γδ T cells express the CCR6 receptor, whose ligand, CCL20, is primarily located on keratinocytes and endothelial cells. The CCR6-CCL20 axis facilitates migration to the site of inflammation, suggesting a role for Vγ4 lymphocytes in this process (135). Similarly, human Vγ2Vγ9 cells have been observed to produce inflammatory mediators such as IFN-γ, IL-17A, TNF-α, CCL3, -4, -5, -6, which are present in psoriasis and aim to recruit immune cells to the skin and stimulate keratinocytes (132). Additionally, these cells are characterized by a high number of scavenger receptors Scart1 and Scart2 on their Surface (133). Scart1 and Scart2 are essential for the function of γδ T cells, especially in skin immunology, allowing these cells to recognize and respond to various ligands, which is crucial for initiating and regulating immune responses and maintaining tissue homeostasis. Moreover, a population of γδ T lymphocytes with high SCART2 expression and low CD5 and CD45RB expression has been identified in the dermis and skin lymph nodes. This group of cells can intensively produce IL-17, which described as a central player in the development of inflammation in psoriasis (136). Approximately 90% of IL-17-producing cells in the dermis are TCR γδ+ cells (137). In a mouse model of psoriasis induced by the Toll-like receptor 7 (TLR7) agonist named imiquimod (IMQ), it was shown that IL-17A-producing Vγ4Vδ4 T cells exhibit similarities, suggesting oligoclonal expansion or a common fetal origin of these cells (138, 139). This may confirm the involvement of SOX (SRY-related HMG-box) transcription factors, which are engaged in embryonic development, in the presence of Vγ4 cells capable of IL-17 production in the dermis (140). However, IL-17 production by γδ T cells requires stimulation via IL-23, which is produced by dermal myeloid dendritic cells and macrophages (137, 141). The IL-23/IL-17 axis exacerbates psoriasis-like conditions, and manipulating IL-23 availability to stimulated cells results in altered IL-17 production (137). Notably, in mice lacking the TCR γδ receptor (TCRδ -/-), IMQ stimulation resulted in significantly attenuated psoriasis-like symptoms, as it limited IL-17 production by dermal cells, rendering IL-17 levels insufficient to initiate acute inflammation (137). In addition to IL-23, IL-1β also influences γδ T cell stimulation. Both IL-1β and IL-23, through IL-1βR and IL-23R receptors on dermal γδ17 T cells, activate intracellular signaling pathways, namely mammalian target of rapamycin (mTOR) and STAT3, respectively. mTOR is a serine/threonine kinase consisting of two complexes, mTORC1 and mTORC2, essential for the survival and proliferation of γδ T lymphocytes, with mTORC2 appearing particularly crucial for dermal γδ17 T cells. The STAT3 pathway, activated via IL-23, is extremely important for the effector functions of murine dermal Vγ4 lymphocytes, while interestingly, Vγ6 cells are STAT3-independent. The mTOR and STAT3 pathways are linked by the transcription factor interferon regulatory factor 4 (IRF4), which regulates *il17* gene expression (142). Various studies indicate that the mTOR and STAT3 pathways are significant for psoriasis development; however, in mouse models, mTORC2, not STAT3, deletion results in a mitigated psoriasis profile and changes in the γδ17 T cells profile (142, 143).

The literature has identified numerous factors controlling the activity of murine dermal γδ T cells and the IL-23/IL-17 axis, influencing the activity and severity of psoriasis. These include the presence of the V-domain immunoglobulin suppressor of T cell activation (VISTA) receptor, Bruton tyrosine kinase (BTK), pro-inflammatory monocytes, IL-36, microRNA molecules such as miR-20 and miR-92b, and complement system molecules (144–149). However, the role of γδ T cells in psoriasis development linked to genes affecting circadian rhythm disruption is of particular interest. Studies by Ando et al. showed that mice with deletions of CLOCK (CLCK; a core circadian gene) and PERIOD2 (PER2; an inhibitor of CLOCK) exhibit mitigated and exacerbated psoriasis symptoms, respectively, after IMQ induction. Importantly, γδ T cells isolated from CLOCK-deficient mice display altered cytokine profiles upon Il-23 stimulation. Moreover, splenic γδ T cells have lower Il-23R -expression since CLOCK binds to the E-box promoter region of the *il23R* gene. Loss of PER2 function yields the exact opposite effects as CLOCK mutation (150). Furthermore, other studies have shown that nuclear receptors Rev-Erb, also involved in circadian regulation, influence the ability of γδ T cells to produce IL-17, thereby mediating psoriasis symptoms (151). These findings suggest a crucial regulatory role of circadian genes in controlling the functionality of γδ T cells in psoriasis.

Evidence indicates that IL-17 produced by dermal γδ T cells is central to the inflammatory response in psoriasis. However, the multitude of factors regulating this process complicates the possibility of control. Therefore, this issue requires continuous research to uncover the functional mechanism and exploit the therapeutic potential of dermal γδ T cells.

### 4.3 Systemic lupus erythematosus

SLE is a chronic autoimmune disease that can affect various organs and tissues in the body, causing a wide range of symptoms such as fatigue, joint pain, skin rashes, and kidney problems. The incidence rate is approximately 40 cases per 100,000 people, with higher prevalence in African American and Hispanic and Caucasian populations (152, 153). The causes of SLE are complex, involving genetic, hormonal, and environmental factors, but this area still requires extensive research (154). Nonetheless, γδ T cells have a complex role in the pathogenesis and regulation of SLE.

Firstly, compared to the control group, the γδ T cell repertoire in SLE patients appears to be quite restricted, primarily involving the Vδ1 and Vδ2 chains, whereas the control group exhibited six active Vδ genes. Additionally, the Vγ9 cells in SLE patients differed from the control group by lacking the EVQEL motif in their junctional sequences and showing limited junctional diversity. Nevertheless, each patient exhibited unique oligoclonal transcripts, with variations in the CDR3 region length, suggesting that γδ T cells undergo peripheral oligoclonal expansion in SLE (155, 156).

Γδ T cells can regulate the humoral response, but they must first be activated through the recognition of autoantigens, such as the chaperonin-containing T-complex protein 1 subunit ζ (CCT6A), whose concentration significantly increases in SLE (156). Then they can be involved in the antibody production process by B cells. Studies on mice have shown that the simultaneous removal of αβ and γδ lymphocytes prevents class-switch recombination and the formation of autoantibodies that exacerbate the disease state in SLE models. It is well known that antibody production is associated with the formation of germinal centers (GCs), where immunoglobulin class switching occurs. This process requires T cells (157), yet evidence from murine studies suggests that γδ T cells play also a significant role in supporting the action of conventional αβ T cells (158). Another study demonstrated that γδ T cells expressing CXCR5 activate CD4+ T cells, which, upon releasing Wnt factors, begin to differentiate into various subtypes, such as T follicular helper (Tfh) cells. Activated CD4+ Tfh cells then migrate to lymphoid follicles in the lymph nodes and spleen, where they encounter naïve B cells and provide the necessary helper signals for GC formation and the initiation of antibody production and class switching (159). TCRγδ-deficient mice show impaired Tfh cell differentiation and GC formation, resulting in lower antibody levels and milder disease symptoms in SLE models (159). When αβ T cells are removed, T-cell-dependent autoantibody production still occurs, suggesting that αβ T cells are not the primary regulators in this process (158, 159).

In the peripheral blood of SLE patients, a decrease in the number of γδ T cells compared to the control group has been observed (160–162) and this decrease was more pronounced in SLE patients with anti-SSB/La antibodies, which occur in about 10-20% of patients (160–163). The level of γδ T cells also inversely correlated with disease activity markers such as SLEDAI, ESR, CRP, and anti-ds-DNA antibodies, which are key diagnostic markers for SLE (160, 161, 164). Additionally, an inverse relationship was found between the level of γδ T cells and the number of circulating plasmablasts, which may contribute to the formation of autoantibodies (164). The decrease in γδ T cells in SLE, even though they seem to be a key regulator of B cells, may be explained by increased apoptosis and reduced proliferation capacity of these cells.

Changes in the cytokine profile produced by peripheral γδ T lymphocytes, which can significantly contribute to the worsening of the patient’s condition, have also been observed (161). As in other autoimmune diseases, IL-17 appears to be a key factor, with its levels markedly increased in SLE patients’ serum (165). Du et al. identified the calcium/calmodulin dependent protein kinase IV (CaMK4) gene, whose expression changes can regulate γδ17 T cell activity in SLE. CaMK4 is a kinase that contributes to the excessive production of IL-17 in lymphatic tissues and kidneys, leading to the mediation of pathological T cell activity and the development of lupus nephritis (LN), which is one of the most common clinical forms of SLE. That suggests possibly a new therapeutic avenue (166).

In summary, γδ T cells play a complex and multifaceted role in the pathogenesis and regulation of SLE, influencing various stages of the immune response and inflammatory process, with a particularly crucial involvement in B cell differentiation and autoantibody production.

### 4.4 Systemic sclerosis

Systemic sclerosis (SSc) is a chronic autoimmune disease characterized by widespread vascular abnormalities and fibrosis of the skin and internal organs. It predominantly affects women and can lead to severe complications involving the lungs, heart, kidneys, and gastrointestinal tract. The pathogenesis of SSc involves a complex interplay between genetic predisposition, environmental factors, and dysregulated immune responses. Understanding the cellular and molecular mechanisms underlying SSc is crucial for developing effective therapeutic strategies and improving patient outcomes (167).

Γδ T cells play a significant role in the pathogenesis of SSc, with their functions and numbers varying depending on the disease stage and their location in the body. In SSc, γδ T cells exhibit limited variability in junctional sequence length regardless of tissue origin, suggesting oligoclonal expression in response to a restricted antigen pool (168). Research indicates a decrease in the total number of γδ T cells in the peripheral blood of SSc patients, especially in the early stages of the disease and in the presence of anti-topoisomerase I antibodies (anti-Scl-70) belonging to anti-nuclear antibodies (ANA) (169, 170). Conversely, there has been a confirmed increase in the Vδ1+ subpopulation in bronchoalveolar lavage fluid (171, 172). In the early phase of SSc, γδ T cells, mainly Vδ1+, also accumulate in perivascular areas of the skin, potentially due to the expression of CD49d (90% of γδ T cells), a ligand for vascular cell adhesion protein (VCAM) involved in lymphocyte migration to tissues. CD49d+γδ T cells were not found in the skin of healthy individuals (172, 173). However, the expression of another cell migration protein, CD62L, is reduced in SSc patients (174).

Γδ T cells also exhibit increased numbers of activating receptors and molecules (HLA-DR, CD69) and cytotoxic mediators (CD8, CD16), potentially exacerbating inflammation and disease severity (172). Additionally, those cells expressing the CD27 receptor show a higher percentage of cytotoxic mediators granzyme B and perforin, indicating their activity and involvement in the pathological state (169). Interestingly, Ueda-Hayakawa reported excessive production of collagen type I alpha 2 chain (COL1A2) mRNA in fibroblasts cultured with γδ T cells (174). However, another study found no differences in the percentage of Vγ9 cells between SSc patients and controls, with the cells maintaining their cytotoxic ability and regulating fibroblast growth through apoptosis induction (175). The discrepancy between these studies may be due to the direct contact between lymphocytes and fibroblasts, leading to different fibroblast response patterns (174, 175).

In terms of cytokine production γδ T cells in SSc exhibit a Th1 polarization, such as IFN-γ production, which contrasts with the Th2 polarization seen in the decidua during pregnancy, where Vδ1 cells inhibit the immune response against the developing fetus (172, 176) This difference suggests the diverse functions of γδ T cells in two distinct conditions, despite the suggestion that γδ T cells interacting with persisting fetal cells post-pregnancy might influence SSc development. These cells can recognize trophoblast antigens, such as Hsp60, and modulate the immune response (176).

The role of γδ T cells in SSc is poorly understood, but existing research suggests it is significant, necessitating further studies.

## 5 Other dysfunctional conditions

### 5.1 Tumor changes

Although tumors are not classified as an immunological disease, they are inextricably linked to the immune system. Due to their unique properties and therapeutic potential, the role of γδ T lymphocytes in tumorigenesis is the subject of intensive research. Γδ T cells have the ability to directly recognize and eliminate cancer cells, making them a key element of the body’s defense against tumor development. Their activity includes cytotoxic mechanisms, interaction with other immune cells, and the production of cytokines that support the immune response and influence the tumor microenvironment. Understanding the role of γδ cells in tumorigenesis may lead to the exploration of novel therapies that will harness these cells against tumor development.

**Figure 3:**
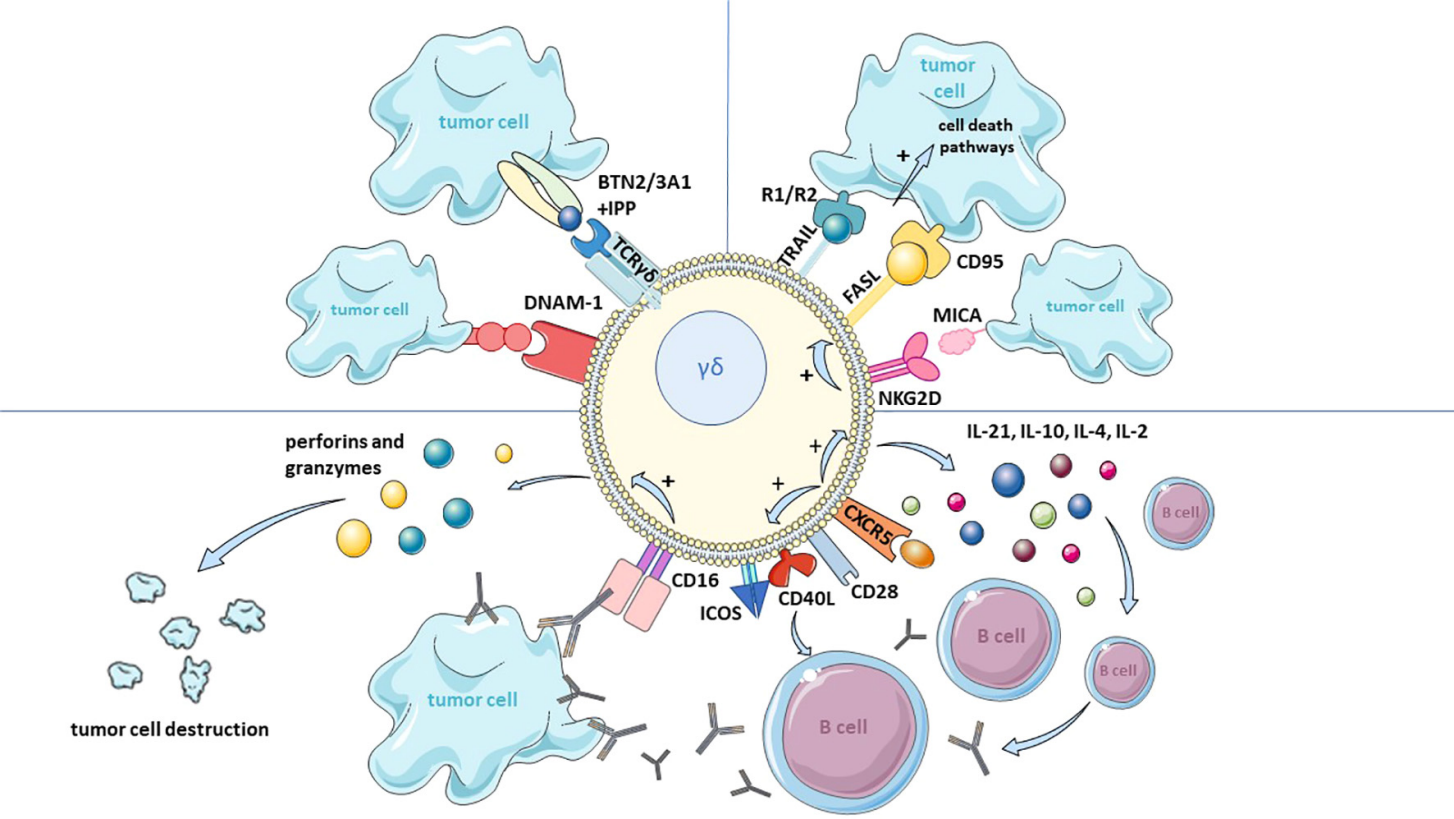
Scheme of γδ T cells interaction with tumor cells aiming their destruction. More detailed description is given in the text. TRAIL, TNF-related apoptosis-inducing ligand; R1/R2, TNF-related apoptosis-inducing receptor; FASL, Fas ligand; CD95, Fas receptor; MICA, MHC class I polypeptide–related sequence A; NKG2D, receptor from the natural killer group 2 D; IL, interleukin; CXCR5, C-X-C motif chemokine receptor; CD28, cluster of differentiation 28, Tp44; CD40L, cluster of differentiation 40, p50; ICOS, inducible T cell costimulatory; CD16, cluster of differentiation, FcγRIII; DNAM-1, DNAX Accessory Molecule-1; BTN2/3A1, butyrophilins 2/3 A1; IPP, isopentenyl pyrophosphate.

The antitumor activity of γδ T cells begins with the recognition of cancerous cells. The γδ TCR is polyclonal; however, its diversity is lower than that of the αβ TCR. This is due to the limited number of V-segment genes and a more restricted clone repertoire in specific tissues. However, this constrained polyclonality, combined with the presence of a longer CDR3 region in the δ chain, allows the γδ TCR to recognize both peptide and non-peptide antigens in an MHC-independent manner. This unique feature is being utilized in the development of novel targeted therapies (177, 178).It is known that the ability of γδ T cells to recognize tumors and initiate direct cytotoxicity against them depends on various receptors and the tumor microenvironment (**Figure 3**). Upon interaction with cancer cells, γδ T cells can express Fas ligand (FASL) and TNF-related apoptosis-inducing ligand (TRAIL), which, upon binding to their respective receptors, Fas (CD95) and TRAIL-R1/R2, activate death pathways in target cells (179, 180). Additionally, FASL induction can be regulated by the activation of NKG2D (181, 182) (**Figure 3**). NKG2D is the best described receptor responsible for cytotoxic activation and recognizes ligands such as MICA, MICB, and ULBP, which are expressed on cancer cell surfaces (183) (**Figure 3**). These ligands are usually absent in healthy tissues, but their expression increases under cellular stress, such as during cancer transformation (184). The TCR γδ also plays a role in tumor recognition. The Vγ9Vδ2 subpopulation can identify phosphoantigens (pAg) such as isopentenyl pyrophosphate (IPP), which are overproduced due to dysregulated mevalonate pathways in cancerous cells (185). Butyrophilin 2A1 (BTN2A1) has emerged as a key molecule in this reaction. BTN2A1, together with BTN3A1, is present on the surface of antigen-presenting cells and cancer cells, and after the internal domain of BTN3A1 binds phosphoantigens, conformational changes in both molecules occur, allowing BTN2A1 to bind to the Vγ9 region of the TCR γδ (186) (**Figure 3**). Studies have shown that BTN2A1 cannot be replaced by any other butyrophilin family molecule, as this leads to the loss of γδ T cell stimulation, while BTN3A1 is essential for pAg recognition (65, 70).

The signal from TCR γδ stimulation is not always sufficient, and in some cases, γδ T cells may require additional receptors, such as CD226 (DNAM-1) or NKp30, to effectively kill cancer cells (187, 188). These diverse receptors allow γδ T cells to flexibly respond to different types of tumors, making them attractive targets in cancer therapy. To date, γδ T cell activity against various cancers, including leukemia, B-cell lymphoma, prostate cancer, melanoma, and mesenchymal glioma, has been described (90, 189–193).

Another mechanism of γδ T cell antitumor activity is antibody-dependent cellular cytotoxicity (ADCC). In this process, γδ T cells participate in recognizing and eliminating tumor cells by binding to antibodies specific for tumor antigens. These antibodies coat the cancer cells, and the Fc fragment of the antibodies binds to the FcγRIII (CD16) receptor on γδ T cells, leading to their activation (194) (**Figure 3**). Upon activation, γδ T cells destroy the coated cancer cells by releasing cytotoxic proteins, such as perforin and granzymes (195). Perforin facilitates the access of proteolytic granzymes to the interior of the target cell, and disruption of these proteins’ functions may result in the sudden onset of cancer (196). Moreover, γδ T cells can enhance ADCC by regulating the maturation and function of B lymphocytes (197). This occurs through a cascade of events, starting with the presence of CXC chemokine receptor type 5 (CXCR5) on γδ T cells, whose stimulation increases the expression of co-stimulatory receptors such as Inducible T-cell costimulator (ICOS), CD40L, and CD28 on Vγ9Vδ2 cells, as well as the production of cytokines IL-21, IL-10, IL-4, and IL-2 (198) (**Figure 3**). The interaction of these receptors with their ligands on B cells (ICOSL, CD40, and CD86) and the action of the produced cytokines stimulate B cells to produce antibodies (195) (**Figure 3**).

Γδ T cells also interact with other cell types, including DCs, which play an essential role in the immune response to tumor formation due to their ability to present tumor antigens (199). DCs stimulation occurs through cytokine signaling as well as direct interactions between the two cell types. Similar to interactions with B cells, CD40, CD80, and CD86 molecules on DCs are involved, and HLA-DR production is stimulated, which is necessary for antigen presentation to T cells (200). Furthermore, γδ T cells can influence the activity of dendritic cells (DCs) through interactions between Toll-like receptors (TLRs) and their ligands on DC’s surface (201). This combined stimulation of γδ T cells and TLRs leads to increased production of pro-inflammatory cytokines, such as IL-12, which promotes Th1-type responses, as demonstrated in co-cultures of the two cell types (202). The effect of DC activation by γδ T cells can be further enhanced by cytokines produced by γδ T cells, particularly IFN-γ, TNF, and IL-6. Unfortunately, γδ T cell cytokine production can also have negative effects in the context of tumorigenesis. The γδ17 T cells subset, capable of producing IL-17, can alter the tumor microenvironment by supporting angiogenesis and the formation of vascular connections that nourish the tumor, a state which is associated with poorer patient outcomes (203, 204).

Research on the role of γδ T lymphocytes in tumorigenesis reveals their crucial role in the immunological control of malignancies. Interactions between cancer cells and the immune system are fundamental for understanding and developing effective therapies. Due to their unique properties, γδ T cells represent a promising direction in cancer immunotherapy. Future studies on their mechanisms of action and potential clinical applications may lead to groundbreaking discoveries that could revolutionize cancer treatment approaches and improve patient outcomes. Thus, γδ T cells are becoming not only the subject of scientific research but also a beacon of hope for new, more effective therapeutic methods in oncology.

## 6 Promising future of therapies based on γδ T cells

With the implementation of Regulation (EU) 536/2014, the regulations governing the registration of trials in Europe have been tightened to ensure transparency at all stages of research (205). It is important to note that as of January 31, 2022, this regulation replaced previous directives to enhance participant protection and facilitate cross-border collaboration in research on new drugs. The Clinical Trials Information System (CTIS) portal and database have been introduced, providing a single location for submitting applications for clinical trial authorization across the European Union (206). Instead of submitting trials in each country individually, researchers can now do so simultaneously for multiple EU member states. All studies had to be transferred to the CTIS system by January 31, 2025 (207).

In the context of clinical research, therapies based on γδ T cells are becoming increasingly important, although they are currently mostly in the research phase and have not yet been fully approved by regulatory authorities such as the FDA in the United States or the EMA in Europe. While they have not reached the status of standard oncological treatment, these therapies are available through clinical trials and orphan drug programs in the United States and many European countries (208, 209).

A search for “gamma delta T cells” in ClinicalTrials.gov (210) yields 41 records of all registered clinical trials under this term. The oldest, registered under NCT01404702, commenced in 2011 and investigated the stimulating effect of zoledronic acid (ZOL) therapy combined with interleukin-2 (IL-2) on γδ T cells capable of killing neuroblastoma cells in a pediatric group. Unfortunately, this study did not yield the expected therapeutic results (211). However, the potential implementation of γδ T cells in this context has not been abandoned. Currently, a phase I study (NCT05400603) is underway in Atlanta (Georgia, United States), aimed to determine the maximum tolerated dose (MTD) and recommended phase II dose (RP2D) of allogeneic expanded γδ T cells in combination therapy for children with refractory or relapsed neuroblastoma and refractory/relapsed osteosarcoma, as well as defining the toxicity of these cells. The sponsor underscores the significance of this research, noting that over half of children suffering from these diseases do not survive or experience treatment-related toxicity. On the other hand, there are reports suggesting potential adverse effects following zoledronic acid treatment, which may lead to the development or recurrence of autoimmune or malignant diseases, possibly involving activated Vγ9δ2 T cells in this process (212). However, further research and observation are needed to confirm these findings.

Current research on γδ T cells focuses on three main categories of therapy: γδ T cell-stimulating therapies, combination therapies with other immune components, and cell engineering therapies utilizing genetically modified γδ T cells. Although these treatment methods have not yet gained full acceptance, they represent a promising direction primarily in oncology (213).

Γδ T cell-stimulating therapies aim to activate, expand, and enhance the natural cytotoxic properties of γδ T cells to improve their ability to recognize and destroy cancer cells. Rather than directly modifying cells, these therapies support their natural activity by stimulating relevant receptors and signaling pathways. This group includes immunotherapy using bisphosphonates, NKG2D agonists, and stimulation with cytokines such as IL-2 and IL-15 (95, 214, 215).

Combination therapies involving γδ T cells with other components of the immune system aim to enhance therapeutic efficacy. The integration of multiple mechanisms enables a more comprehensive and effective immune response. Examples include therapies based on monoclonal antibodies, where γδ T cells cooperate with antibodies such as rituximab to strengthen the ADCC response; combinations with TLR modulators that influence γδ T cell activity towards DCs, as well as combination therapies with checkpoint inhibitors, including blocking PD-1 and CTLA-4 receptors (216–219).

**Figure 4:**
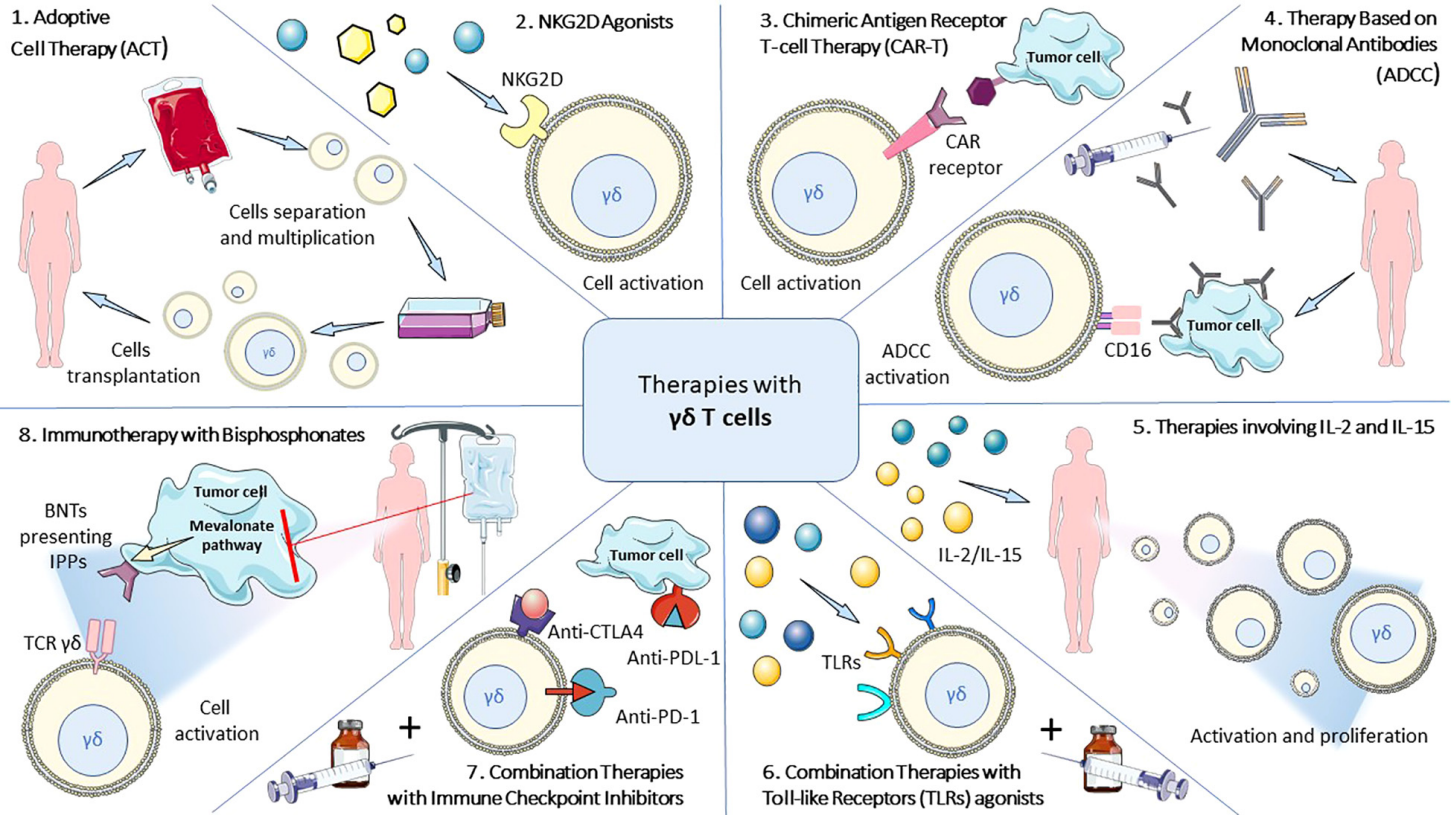
Scheme of novel therapeutic regimens utilizing γδ T cells with therapeutic potential.

A novel concept involves the utilization of genetic engineering methods to introduce changes into γδ T cells (**Figure 4**). This therapy involves genetically modifying γδ T cells equipped with specific receptors (e.g., CAR) (220) that enable more precise and effective recognition of cancer cells. As a result, γδ T cells can target tumors more accurately. The classic CAR-T therapy was approved by the FDA in 2017 for treating B-cell acute lymphoblastic leukemia and diffuse large B-cell lymphoma (DLBCL) (221). The procedure is based on the classical Adoptive Cell Therapy (ACT) method, which involves collecting T cells from the patient, expanding them *in vitro*, and then reinfusing them into the patient, with the added aspect of genetic modification in CAR therapy. Another option is the introduction of allogeneic modified γδ T cells from donors into the patient’s body (222).

As of the writing of this article (September 2024), three active clinical trials regarding CAR-T γδ therapies are visible in the ClinicalTrials.gov database. The oldest, which is actively recruiting patients, concerns the safety and efficacy of therapy in patients suffering from severe SLE (NCT06106893) (223). Two additional studies, registered in the second half of 2024, are also Phase I or I/II trials, concerning lupus nephritis (NCT06375993) and clear cell renal cell carcinoma (ccRCC) (NCT06480565). A notable difference in these clinical trials is the functionality of CAR-Tγδ cells, as they target various antigens characteristic of the diseases. In the study NCT06375993, the target antigen is CD20, while for NCT06480565, it is CD70 (224, 225).

In addition to registered CAR-Tγδ clinical trials, there are 10 active studies concerning other therapies using γδ T cells. Most of these involve allogeneic γδ T cells administered to patients with various types of solid tumors (NCT04765462, NCT05400603), glioblastoma multiforme (GBM) (NCT05664243), and blood cancers (NCT053588, NCT04764513). The tested methods pertain to both monotherapy and combination therapies with traditional oncological treatment methods. Furthermore, an anti-PD1 therapy using γδ T cells for solid tumors is also in the research phase (NCT06404281), along with allogeneic γδ T cells transplants (NCT06364787, NCT06364800, NCT06212388).

A particularly noteworthy investigation is the application of bispecific antibodies targeting anti-Vδ2 and anti-prostate specific membrane antigen (PSMA) for the treatment of metastatic castration resistant prostate cancer (mCRPC). This innovative therapy aims to enhance the cytotoxic response of γδ T cells against tumor cells (NCT05369000) (226). The study is set to conclude by the end of 2027, and its findings may be of value given that another drug from the same sponsor, LAVA-051, which is also a bispecific antibody, has been approved by the FDA as an orphan drug for treating chronic lymphocytic leukemia (CLL) (227). Nevertheless, according to the clinicaltrials.gov database (accessed on March 17, 2025), the study was terminated due to business reasons, not related to product safety issues.

The utilization of γδ T cell-based therapies in autoimmune diseases, including rheumatological disorders, remains in its infancy. There is still a substantial need for further research and collaboration among the scientific and medical communities to make such treatments feasible and accessible. Nonetheless, the scientific landscape is continuously evolving, providing new evidence that underscores the significance of this area of inquiry. The dualistic nature of γδ T cells presents considerable immunomodulatory and therapeutic potential, which merits further exploration and attention (228–230).

## 7 Conclusions

γδ T cells are becoming an increasingly intriguing area of research, particularly due to their therapeutic potential in the treatment of autoimmune diseases and cancers. With their unique properties, γδ T cells demonstrate the ability to quickly recognize and eliminate cancer cells, positioning them as promising candidates in cancer immunotherapy. Studies suggest that these cells can act as regulators of immune responses, which may be crucial in the context of autoimmune diseases where the balance between pro-inflammatory and anti-inflammatory responses is disrupted.

It is also noteworthy that γδ T cells possess the ability to support other immune cells, enhancing their therapeutic efficacy. Their dualistic action allows them to be both pro-inflammatory and anti-inflammatory, creating opportunities for developing personalized therapies tailored to the specific needs of patients. Further research into the mechanisms of γδ T cell action may reveal new treatment strategies that could benefit patients who currently lack effective therapeutic options.

Clinical trial examples involving γδ T cell therapies indicate that their potential in clinical practice is significant. While these therapies are still in the research phase, the results of previous clinical experiences suggest that γδ T cells may play a pivotal role in future immunotherapeutic approaches. Given the growing interest in new strategies for cancer and autoimmune disease treatment, it is worthwhile to continue exploring γδ T cells to fully understand their capabilities and potential clinical applications.
